# Investigation of the association between the genetic polymorphisms of the co-stimulatory system and systemic lupus erythematosus

**DOI:** 10.3389/fimmu.2022.946456

**Published:** 2022-09-06

**Authors:** Ding-Ping Chen, Wei-Tzu Lin, Kuang-Hui Yu

**Affiliations:** ^1^ Department of Laboratory Medicine, Linkou Chang Gung Memorial Hospital, Taoyuan, Taiwan; ^2^ Department of Medical Biotechnology and Laboratory Science, College of Medicine, Chang Gung University, Taoyuan, Taiwan; ^3^ Division of Rheumatology, Allergy, and Immunology, Chang Gung University and Memorial Hospital, Taoyuan, Taiwan

**Keywords:** systemic lupus erythematosus (SLE), co-stimulatory/co-inhibitory molecules, single nucleotide polymorphism (SNP), autoimmune disease (AD), association

## Abstract

Human leukocyte antigen genes have been shown to have the strongest association with autoimmune disease (AD). However, non-HLA genes would be risk factors of AD. Many genes encoding proteins that are related to T- and B-cell function have been identified as susceptibility genes of systemic lupus erythematosus (SLE). In this study, we explored the correlation between SLE and the genetic polymorphisms of co-stimulatory/co-inhibitory molecules, including CTLA4, CD28, ICOS, PDCD1, and TNFSF4. We found that there were nine single-nucleotide polymorphisms (SNPs) associated with SLE, namely, rs11571315 (TT vs. CT vs. CC: p < 0.001; TT vs. CT: p = 0.001; p = 0.005; TT vs. CT +CC: p < 0.001; TT+CT vs. CC: p = 0.032), rs733618 (CC vs. CT vs. TT: p = 0.002; CC vs. CT: p = 0.001; CC vs. TT: p = 0.018; CC vs. CT + TT: p = 0.001), rs4553808 (AA vs. AG: p < 0.001), rs62182595 (GG vs. AG vs. AA: p < 0.001; GG vs. AG: p < 0.001; GG vs. AG+AA: p < 0.001), rs16840252 (CC vs. CT vs. TT: p < 0.001; CC vs. CT: p < 0.001; CC vs. CT + TT: p < 0.001), rs5742909 (CC vs. CT: p = 0.027; CC vs. CT + TT: p = 0.044), rs11571319 (GG vs. AG vs. AA: p < 0.001, GG vs. AG: p < 0.001; GG vs. AG+AA: p < 0.001), rs36084323 (CC vs. CT vs. TT: p = 0.013, CC vs. TT: p = 0.004; CC vs. CT + TT: p = 0.015; CC +CT vs. TT: p = 0.015), and rs1234314 (CC vs. CG vs. GG: p = 0.005; CC vs. GG: p=0.004; CC+ CG vs. GG: p=0.001), but not in CD28 and ICOS by using the chi-square test. Additionally, rs62182595 and rs16840252 of CTLA and rs1234314 and rs45454293 of TNFSF4 were also associated with SLE in haplotypes. These SLE-related SNPs also had an association with several diseases. It was indicated that these SNPs may play an important role in immune regulation and pathogenic mechanisms.

## Introduction

Autoimmune diseases (ADs) are a heterogeneous group of diseases that involve the connective tissues, skin, subcutaneous tissues, muscles, joints, and various internal organs. In a Taiwanese population, the incidence of systemic lupus erythematosus (SLE) was 7.2 per 100,000 person-years (1), particularly affecting the women of childbearing age. Because SLE is a complex and multifactorial disorder, its course is difficult to accurately judge. If AD cannot be treated at the first time, it will lead to poor prognosis. Moreover, data showed that the heritability of SLE was up to 43.9% (2). Thus, we tried to investigate the correlation between genetic polymorphism and SLE.

SLE is a multisystem autoimmune disease characterized by the production of autoantibodies. The inappropriate T‐cell‐dependent expansion of autoreactive B cells is considered to play a role in the production of pathogenic autoantibodies against autoantigens (3). The pathogenesis of SLE is still unclear yet. An increasing number of studies showed that the overactivation of autoreactive T cells is the main factor in AD (4, 5). In general, the autoreactive T cells that can recognize the self-antigen will go to apoptosis through positive and negative selection in the thymus (6). However, even if such a rigorous selection mechanism is complied, a small number of T cells escape into the peripheral blood after the selection, in which these escaped T cells will recognize self-antigens and attack to own cells and tissues. In the peripheral blood system, autoreactive T cells are regulated by regulatory T cells (Treg). When these autoreactive T cells cannot be controlled by Treg, also known as loss of immune tolerance, it will lead to become one of the main causes of autoimmune diseases.

To date, it has been found that human leukocyte antigen (HLA) genes generally have the strongest association with AD (7). However, other genes located outside of the HLA region may be a risk factor of AD. Several genes involved in the etiology of SLE have been widely studied, and the single-nucleotide polymorphisms (SNPs) of these genes encoding proteins related to T- and B-cell functions have been considered as the susceptibility loci of SLE. Studies showed that T-cell activation was strictly regulated by signals from co-stimulatory and co-inhibitory molecules (8). Among them, the most popular hot genes are cytotoxic T-lymphocyte-associated protein 4 (CTLA4) and CD28. CD28 is continuously expressed on naïve and mature T cells. After binding with CD80/CD86 on antigen-presenting cells (APCs), it provides a stimulatory signal to promote activation of T cells, giving them the ability to attack (9).

CTLA4 is a co-inhibitory molecule, which is induced to express on the surface of T cells after CD28 interacts with CD80/CD86 on APCs and stimulates T-cell activation. By competing with CD28 for CD80/CD86, it plays a role in inhibition of T-cell activation. Thus, the balance between CTLA4 and CD28 is an important key for T-cell activation or inhibition (10). Both programmed cell death protein 1 (PDCD1; PD1) and CTLA4 are immune checkpoints. When the PD1 protein binds to its ligand on APCs, it will induce the activation of an immune-receptor tyrosine-based inhibitory motif (ITIM) in the cytoplasmic tail of PD1, so as to inhibit the activation of T cells (11). Inducible co-stimulator (ICOS), like CD28, acts as a co-stimulatory receptor for T cells. It is essential for the activation of T cells and further promotes the humoral immune response (12). In addition, it was found that ICOS was highly expressed on T cells of SLE patients (13). When tumor necrosis factor superfamily member 4 (TNFSF4; OX40L) binds to its receptor (OX40), it can also provide a signal to promote T-cell activation, and studies have shown that OX40L can stimulate T-cell response and promote the pathogenesis of SLE (14). Therefore, these five genes were considered in the present study. Moreover, it is worth noting that the susceptible SNP of the disease has ethnic variations. Therefore, the aim of this study was to find out the SLE susceptibility loci of a Taiwanese population and apply it to the clinic to assist physicians in diagnosis.

## Methods and material

### Study subjects

The Institutional Review Board of Chang Gung Memorial Hospital has reviewed and approved the study. The approval ID was 202002097B0. All study subjects signed informed consent and performed in accordance with relevant guidelines and regulation. There were 75 SLE patients who participated in the present study, in which the average onset age was 32.99 ± 1.49, and there were 66 women (88%) and nine men (12%). The inclusion criteria for SLE were based on the diagnostic criteria established by the American College of Rheumatology: 1) malar rash, 2) discoid lupus erythematosus with desquamation, 3) UV sensitivity on skin, 4) oral ulcer, 5) non-erosive arthritis, 6) serositis, pleurisy, or pericarditis, 7) kidney lesions, 8) nervous system lesions, 9) hematological lesions, 10) immunological lesions, 11) positive antinuclear antibody (ANA) test. If four of the 11 items are met, they can be diagnosed as SLE. In the definition of laboratory diagnosis, it is mainly to detect autoantibodies. ANA positivity exists in more than 95% of SLE patients. However, ANA will increase with age, and normal people may also have positive results, so the clinically meaningful results of ANA need to be ≥1:160x. Furthermore, 75 volunteers without immune abnormalities were collected for a case–control study, in which the average age was 31.83 ± 0.93 and there were 60 women (80%) and 15 men (20%).

### DNA extraction and sequencing

Three milliliters of peripheral blood of autoimmune disease patients and healthy controls was collected in EDTA-coated vacuum tubes, and QIAamp DNA Blood Mini Kit (Qiagen, Valencia, California, USA) was used to extract the genomic DNA. After PCR reaction, the concentration and purity of extracted DNA were measured using a UV spectrometer. The PCR mixture contained 50 ng of DNA, 7.5 µl of HotStarTaq DNA Polymerase (Qiagen GmbH, Hilden, Germany) or 2X Tag polymerase, each 1 µl of forward and reverse primers (10 μM), and 14.5 µl of ddH_2_O. The primer pairs of each gene region and the PCR programs are shown in **Table 1**. Then, the BigDye Terminator Cycle Sequencing Kit (Thermo Fisher, Waltham, Massachusetts, USA) and the ABI PRISM Genetic Analyzer (Thermo Fisher, Waltham, Massachusetts, USA) were used for direct sequencing according to the manufacturer’s instructions.

**Table 1:** The pair primers used for amplifying the DNA fragments in this study.

Gene	Region	Sequence
CTLA4	Promoter	F: 5′ GGCAACAGAGACCCCACCGTT 3′
		R: 5′ GAGGACCTTCCTTAAATCTGGAGAG 3′
	95°C 10 min, 35[94°C 30 sec, 65.5°C 30 sec, 72°C 60 sec] 72°C 3 min	
	Promoter-exon1	F:5′ CTCTCCAGATTTAAGGAAGGTCCTC 3′
		R: 5′ GGAATACAGAGCCAGCCAAGCC 3′
	95°C 10 min, 35[94°C 30 sec, 65.5°C 30 sec, 72°C 60 sec] 72°C 3 min	
	Exon4-3′UTR	F: 5′ CTA GGG ACC CAA TAT GTG TTG 3′
		R: 5′ AGA AAC ATC CCA GCT CTG TC 3′
	95°C 10 min, 35[94°C 30 sec, 59°C 30 sec, 72°C 60 sec] 72°C 3 min	
CD28	Promoter	F: 5′- GGG TGG TAA GAA TGT GGA TGA ATC-3′
		R: 5′-CAA GGC ATC CTG ACT GCA GCA-3′
	95°C 3 min, 30[95°C 30 sec, 58°C 30 sec, 72°C 120 sec] 72°C 3 min	
	Intron3	F: 5′- AAG GAT GCA GTT TAG GGT CTA GAT T -3′
		R: 5′-GAT CAA GCC AAC ATT GTC CAT TGG-3′
	95°C 3 min, 30[95°C 30 sec, 58°C 30 sec, 72°C 120 sec] 72°C 3 min	
PDCD1	Promoter-exon1	F: 5′- AAAC TGA GGG TGG AAG GTC CCT-3′
		R: 5′- ACC CAC ACA GCC TCA CAT CTC T -3′
	95°C 10 min, 35[94°C 30 sec, 55°C 30 sec, 72°C 60 sec] 72°C 7 min	
	Intron4-exon5	F: 5′-GCC TGT GTG TTT CTG GGA CAG-3′
		R: 5′-AGC GCA TTT CCT CAG GAG AAG C-3′
	95°C 3 min, 35[95°C 30 sec, 61°C 30 sec, 72°C 120 sec] 72°C 10 min	
	Exon5-3′UTR	F: 5′-ATC TCC AAC CAG CCC CCA AGT T-3′
		R: 5′-TGC AGG GAC AAT AGG AGC CAG-3′
	95°C 3 min, 35[95°C 30 sec, 61°C 30 sec, 72°C 120 sec] 72°C 10 min	
ICOS	Promoter	F:5′-GTCAATTGTTCTCCACTGCCTGCC-3′
		R:5′-GGTGCTCCAGAGATAAGAAGAAAGCCTTTG-3′
	Promoter	F:5′-CTCTGCTGTAATATATGAGGAGCAGGG-3′
		R:5′-CACTGACAGGTAACTCCAAGCAGG-3′
	95°C 10 min, 36[95°C 60 sec, 60°C 60 sec, 72°C 60 sec] 72°C 10 min	
	Exon5-3′UTR	F:5′-GTAGGGAACTGGCACATGGAGAG-3′
		R:5′-GATAAGTGGCTCCTCTTAAAACTGG-3′
	95°C 3 min, 30[95°C 30 sec, 58°C 30 sec, 72°C 120 sec] 72°C 3 min	
TNFSF4	Promoter-exon1	F: 5′-GGCTTGGAGTCTATGATATTGTGCC-3′
		R: 5′-GAAGGGCGTTTAACCACACTTTACG-3′
	95°C 10 min, 36[95°C 60 sec, 60°C 60 sec, 72°C 60 sec] 72°C 10 min	

F, forward primer; R, reverse primer; min, minutes; sec, seconds.

### Selection of candidate SNPs

Because the abnormal expression level of co-stimulatory/co-inhibitory molecules may contribute to autoimmune diseases (15), and the SNP variation located in the promoter region may alter the expression level of mRNA expression, we focused on the promoter region to look for the SNPs related to SLE. In addition, we also searched the SNPs related to autoimmune diseases that were published in previous studies through NCBI, including rs4404254 and rs4675379 located in the three prime untranslated regions (3′UTR) of ICOS gene (16), rs2227982 located in exon 5 and rs10204525 located in the 3′UTR of PDCD1 gene (17), rs3087243 located in the 3′UTR of CTLA4 gene (18), and rs3116496 located in the intron 3 of CD28 gene (19). These SNPs were selected as candidate SNPs, and the region from the 500 bp upstream to 500 bp downstream of these candidate SNPs was amplified, so as to find the SNPs related to SLE in the Taiwanese population.

### Statistical analysis

All the allele frequencies of each SNP in the control group were in accordance with the Hardy–Weinberg equilibrium (HWE) (**Table 2**). The allele frequency and genotype frequency between patients and healthy controls were determined by chi-squared test and Fisher’s exact test and were given an odds ratio with a 95% confidence interval. These statistical data were calculated by SPSS 17.0. D′ was used to estimate the linkage disequilibrium (LD) by comparing the observed and expected frequencies of one haplotype involved in alleles at different loci. The block was defined as it scarcely had evidence for historical recombination in this region (20). The figures of LD were produced by Haploview 4.2 (https://www.broadinstitute.org/haploview/haploview).

**Table 2:** The HWE analysis in control group and the allele frequencies in cases and controls.

SNP	Position	Allele	Minor allele frequency		HWE *p* value	Odds ratio	*p^a^* value
			Patient	Control		(95%CI)	
CTLA4							
rs11571315	203866178	**C**/T	0.148	0.353	0.710	0.318 (0.179-0.563)	<0.001*
rs733618	203866221	T/**C**	0.417	0.573	0.817	0.532 (0.335-0.845)	0.007*
rs4553808	203866282	A/**G**	0.007	0.133	0.412	0.045 (0.006-0.343)	<0.001*
rs11571316	203866366	**A**/G	0.157	0.220	0.654	0.661 (0.364-1.201)	0.172
rs62182595	203866465	**A**/G	0.007	0.133	0.946	0.047 (0.006-0.353)	<0.001*
rs16840252	203866796	C/**T**	0.021	0.147	0.330	0.126 (0.037-0.430)	<0.001*
rs5742909	203867624	C/**T**	0.079	0.140	0.370	0.524 (0.243-1.130)	0.095
rs231775	203867991	**A**/G	0.325	0.349	0.999	0.899 (0.542-1.488)	0.678
rs3087243	203874196	G/**A**	0.239	0.227	0.752	1.013 (0.589-1.740)	0.964
rs11571319	203874215	G/**A**	0.132	0.280	0.814	0.358 (0.196-0.655)	0.001*
CD28							
rs1879877	203705277	G/**T**	0.466	0.456	0.991	1.083 (0.684-1.714)	0.733
rs3181096	203705369	C/**T**	0.247	0.284	0.220	0.826 (0.494-1.383)	0.468
rs3181097	203705416	G/**A**	0.419	0.419	0.895	1.000 (0.630-1.587)	1.000
rs3181098	203705655	G/**A**	0.277	0.258	0.164	1.109 (0.662-1.857)	0.693
rs56228674	203729436	C/**T**	0.033	0.040	0.979	0.828 (0.155-4.405)	1.000
rs3116496	203729789	T/**C**	0.107	0.120	0.793	0.876 (0.323-2.376)	0.794
PDCD1							
rs5839828	241859601	**G**/GG	0.338	0.289	0.868	1.258 (0.761-2.079)	0.371
rs36084323	241859444	**C**/T	0.493	0.317	0.997	2.096 (1.293-3.397)	0.003*
rs41386349	241851697	G/**A**	0.222	0.180	0.470	1.302 (0.734-2.308)	0.366
rs6705653	241851407	**T**/C	0.285	0.216	0.572	1.443 (0.847-2.459)	0.177
rs2227982	241851281	**G**/A	0.471	0.392	0.953	1.384 (0.867-2.210)	0.173
rs2227981	241851121	**A**/G	0.261	0.223	0.297	1.232 (0.713-2.127)	0.454
rs10204525	241850169	**C**/T	0.250	0.207	0.990	1.280 (0.642-2.552)	0.483
ICOS							
rs11571305	203935403	G/**A**	0.297	0.336	0.007*	0.836 (0.504-1.388)	0.489
rs11889352	203935948	**T**/A	0.254	0.243	0.126	1.059 (0.617-1.818)	0.836
rs11883722	203936122	G/**A**	0.418	0.421	0.491	0.985 (0.616-1.576)	0.951
rs10932029	203937045	T/**C**	0.164	0.110	0.350	1.586 (0.789-3.188)	0.193
rs10932035	203959929	**G**/A	0.463	0.500	<0.001*	0.833 (0.486-1.430)	0.508
rs10932036	203960458	**A**/T	0.047	0.056	0.154	0.844 (0.288-2.475)	0.757
rs4404254	203960563	T/**C**	0.192	0.269	0.995	0.646 (0.368-1.133)	0.126
rs10932037	:203960623	C/**T**	0.034	0.082	0.673	0.397 (0.134-1.173)	0.085
rs10932038	203960861	A/**G**	0.035	0.077	0.561	0.432 (0.144-1.298)	0.125
rs1559931	203961006	G/**A**	0.197	0.227	0.980	0.838 (0.467-1.505)	0.555
rs56259923	203961015	G/**T**	0.014	0.016	0.992	0.900 (0.125-6.484)	1.000
rs4675379	203961372	G/**C**	0.156	0.161	0.598	0.967 (0.393-2.381)	0.942
TNFSF4							
rs1234314	173208253	G/**C**	0.514	0.360	0.395	1.881 (1.177-3.005)	0.008*
rs45454293	173208097	C/**T**	0.148	0.160	0.998	0.911 (0.482-1.722)	0.774

The position was obtained from Genome Assembly GRCh38.p13. rs: reference SNP; HWE: Hardy-Weinberg equilibrium; 95% CI: 95% confidence interval; P^a^ values of allele frequency were counted from Chi-square test or Fisher’s exact test. In the column of “Allele”, the bold was referred to minor allele, and the minor allele was referred to the allele with lower frequency in the population containing cases and controls. “*” was expressed as p<0.05.

## Results

### Hardy–Weinberg equilibrium test

The genotype frequencies based on controls were checked for Hardy–Weinberg equilibrium (HWE). It was found that the rs11571305 and rs10932035 of ICOS violated HWE, which indicated that these SNPs were not sufficient to represent the genotype distribution of the population, so the subsequent analysis results of these SNPs were less credible (**Table 2**). Thus, these SNPs were not analyzed and discussed.

### The analysis of allele frequencies

The allele frequencies of the six SNPs in the CTLA4 gene were significantly different between cases and controls, including those of rs11571315 (p < 0.001), rs733618 (p = 0.007), rs4553808 (p < 0.001), rs62182595 (p < 0.001), rs16840252 (p < 0.001), and rs11571319 (p = 0.001). For the subjects with a minor allele at these six loci, they had a lower risk (0.045–0.532 times) of SLE. Moreover, in the PDCD1 gene and TNFSF4 gene, each SNP had significance between the SLE group and control group, including rs36084323 (p = 0.003) and rs1234314 (p = 0.008). For the subjects with a minor allele at these two loci, they had a higher risk (1.881 and 2.096 times, respectively) of SLE (**Table 2**).

### The analysis of genotype frequencies

There were seven SNPs in the CTLA4 gene, one SNP in the PDCD1 gene, and one SNP in the TNFSF4 gene that had statistical significance (**Table 3**). The genotype frequency (CC vs. CT vs. TT) of rs11571315 was significantly different between cases and controls (p < 0.001). Compared to TT, the subjects with a CT genotype at rs11571315 would have a lower risk of SLE (OR = 0.301, 95% CI = 0.142–0.641, p = 0.001), and those with CC would have a 0.17 times risk of SLE (95% CI = 0.044–0.654, p = 0.005). Additionally, the genotype frequencies were analyzed through the dominant model (AA vs. Aa +aa) and recessive model (AA +Aa vs. aa), where “A” was referred to as the allele with higher frequency in the population, also known as major allele. Based on the dominant model, the subjects who had at least one C allele (CT+CC) at rs11571315 would have a 0.267 times risk of SLE (95% CI = 0.132–0.539, p < 0.001), which also had significance based on the recessive model. The subjects with the CC genotype would have a lower risk of SLE (OR = 0.257, 95% CI = 0.068–0.962, p = 0.032) than those with at least one T-allele (TT+CT). The genotype frequency (CC vs. CT vs. TT) of rs733618 had significance (p = 0.002). Compared to CC, the subjects with the CT genotype would have a lower risk of SLE (OR = 0.241, 95% CI = 0.104–0.555, p = 0.001), and those with TT would have a 0.367 times risk of SLE (95% CI = 0.159–0.849, p = 0.018), which also had significance based on the dominant model (CC vs. CT+TT: OR = 0.295, 95% CI = 0.142–0.614, p = 0.001). The genotype frequency (AA vs. AG) of rs4553808 had significance (p < 0.001), in which there was no subject with the GG genotype at this locus. Compared to the AA genotype, the subjects with AG would have a lower risk of SLE (OR = 0.039, 95% CI = 0.005–0.298, <0.001). The genotype frequency (GG vs. AG vs. AA) of rs62182595 had significance (p < 0.001). Compared to GG, the subjects with the AG genotype would have a lower risk of SLE (OR = 0.045, 95% CI = 0.006–0.348, p < 0.001), which also had significance based on the dominant model (GG vs. AG+AA: OR = 0.043, 95% CI = 0.006–0.329, p < 0.001). The genotype frequency (CC vs. CT vs. TT) of rs16840252 was significantly different between cases and controls (p < 0.001). Compared to the CC genotype, the subjects with the CT genotype would have a 0.035 times risk of SLE (95% CI = 0.005–0.267, p < 0.001), which also had significance based on the dominant model (CC vs. CT+TT: OR = 0.070, 95% CI = 0.016–0.310, p < 0.001). The genotype frequency of rs5742909 had a strong tendency toward statistical significance (p = 0.051). Compared to the CC genotype, the subjects with the CT genotype would have a 0.386 times risk of SLE (95% CI = 0.163–0.914, p = 0.027), which also had significance based on the dominant model (CC vs. CT + TT: OR = 0.429, 95% CI = 0.185–0.991, p = 0.044). The genotype frequency (GG vs. AG vs. AA) of rs11571319 had significance (p < 0.001). Compared to GG, the subjects with the AG genotype would have a 0.049 times risk of SLE 95% CI = 0.011–0.219, p < 0.001), which also had significance based on the dominant model (GG vs. AG+AA: OR = 0.197, 95% CI = 0.088–0.443, p < 0.001). The genotype frequency (CC vs. CT vs. TT) of rs36084323 was significantly different between cases and controls (p = 0.013). Compared to the CC genotype, the subjects with the TT genotype would have a 4.466 times risk of SLE (95% CI = 1.579–12.631, p = 0.004), which also had significance based on the dominant model (CC vs. CT+TT: OR = 2.377, 95% CI = 1.177–4.798, p = 0.015) and recessive model (CC +CT vs. TT: OR = 3.105, 95% CI = 1.206–7.996, p = 0.015). The genotype frequency (CC vs. CG vs. GG) of rs1234314 was significantly different between cases and controls (p = 0.005). Compared to the CC genotype, the subjects with the GG genotype would have a 4.4 times risk of SLE (95% CI =1.577-12.275, p = 0.004), which also had significance based on the dominant model (CC+CG vs. GG: OR = 4.362, 95% CI = 1.727-11.015, p = 0.001).

**Table 3:** Genotype frequencies of the significant SNPs in SLE cases and healthy controls.

SNP	Genotype	Genotype frequency		Odds ratio 95% CI.	*p* value
		Patient	Control		
CTLA4					
rs11571315	CC vs. CT vs. TT				0.001*
	TT	53	33	Ref.	1.000
	CT	15	31	0.301 (0.142-0.641)	0.001
	CC	3	11	0.170 (0.044-0.654)	0.005**
	TT vs. CT + CC			0.267 (0.132-0.539)	<0.001*
	TT + CT vs. CC			0.257 (0.068-0.962)	0.032*
rs733618	CC vs. CT vs. TT				0.002*
	CC	33	15	Ref.	1.000
	CT	18	34	0.241 (0.104-0.555)	0.001*
	TT	21	26	0.367 (0.159-0.849)	0.018*
	CC vs. CT + TT			0.295 (0.142-0.614)	0.001*
	CC + CT vs. TT			0.776 (0.387-1.556)	0.475
rs4553808	AA vs. AG vs. GG				<0.001*
	AA	71	55	Ref.	1.000
	AG	1	20	0.039 (0.005-0.298)	<0.001*
	GG	0	0	NA	NA
	AA vs. AG+GG			0.039 (0.005-0.298)	<0.001*
	AA+AG vs. GG			NA	NA
rs62182595	GG vs. AG vs. AA				<0.001*
	GG	69	56	Ref.	1.000
	AG	1	18	0.045 (0.006-0.348)	<0.001**
	AA	0	1	NA	0.452
	GG vs. AG+AA			0.043 (0.006-0.329)	<0.001*
	GG+AG vs. AA			NA	1.000
rs16840252	CC vs. CT vs. TT				<0.001*
	CC	69	53	Ref.	1.000
	CT	1	22	0.035 (0.005-0.267)	<0.001*
	TT	1	0	NA	1.000
	CC vs. CT + TT			0.070 (0.016-0.310)	<0.001*
	CC + CT vs. TT			NA	0.486
rs5742909	CC vs. CT vs. TT				0.051
	CC	60	54	Ref.	1.000
	CT	9	21	0.386 (0.163-0.914)	0.027*
	TT	1	0	NA	1.000
	CC vs. CT + TT			0.429 (0.185-0.991)	0.044*
	CC + CT vs. TT			NA	0.493
rs11571319	GG vs. AG vs. AA				<0.001*
	GG	58	40	Ref.	1.000
	AG	2	28	0.049 (0.011-0.219)	<0.001*
	AA	8	7	0.788 (0.265-2.348)	0.669
	GG vs. AG+AA			0.197 (0.088-0.443)	<0.001*
	GG+AG vs. AA			1.295 (0.443-3.784)	0.636
PDCD1					
rs36084323	CC vs. CT vs. TT				0.013*
	TT	19	33	Ref.	1.000
	CT	34	31	1.905 (0.904-4.014)	0.089
	CC	18	7	4.466 (1.579-12.631)	0.004*
	TT vs. CT+CC			2.377 (1.177-4.798)	0.015*
	TT+CT vs. TT			3.105 (1.206-7.996)	0.015*
TNFSF4					
rs1234314	CC vs. CG vs. GG				0.005*
	CC	20	28	Ref.	1.000
	CG	29	40	1.015 (0.481-2.142)	0.969
	GG	22	7	4.400 (1.577-12.275)	0.004*
	CC vs. CG+ GG			1.519 (0.756-3.051)	0.239
	CC+ CG vs.GG			4.362 (1.727-11.015)	0.001*

95% CI, 95% confidence interval; NA, not applicable. “*” was expressed as p<0.05.

### Linkage disequilibrium and haplotype analysis

**Figure 1:**
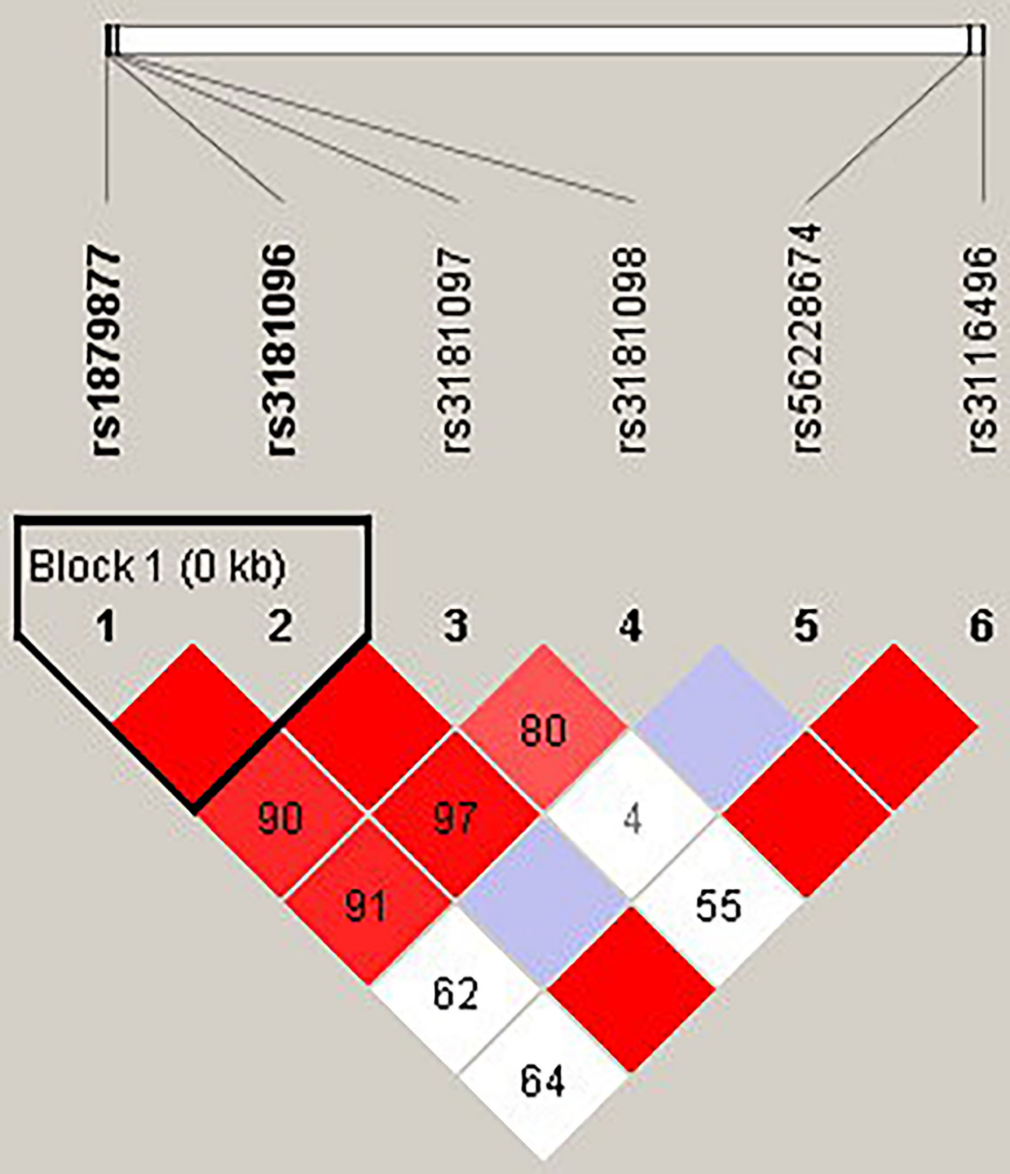
The linkage disequilibrium (LD) analysis of the CD28 gene. There was one haplotype block shown in the CD28 gene, including rs1879877 and rs3181096.

**Figure 2:**
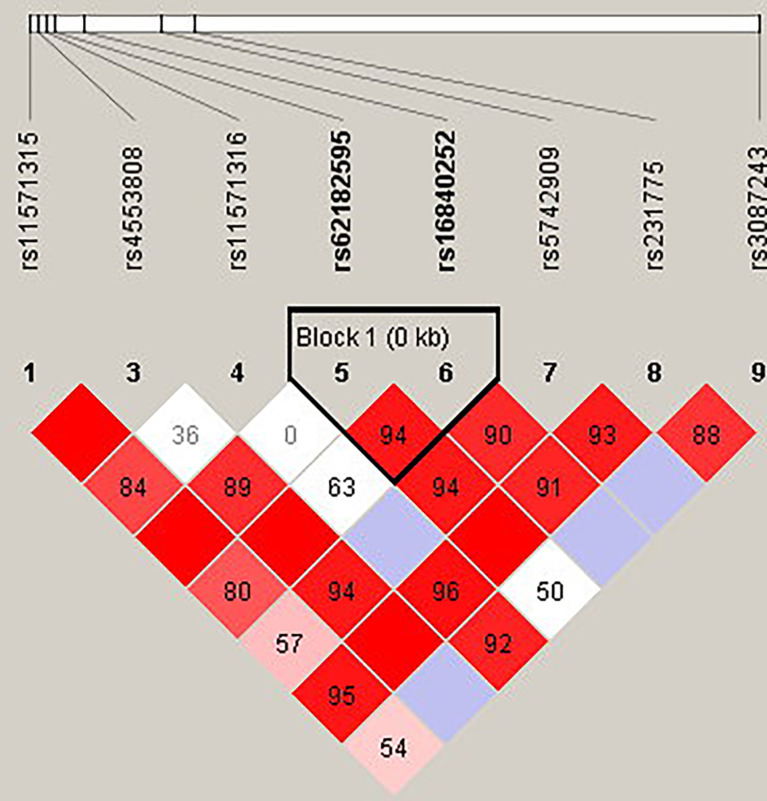
The linkage disequilibrium (LD) analysis of the CTLA4 gene. There was one haplotype block shown in the CTLA4 gene, including rs62182595 and rs16840252.

**Figure 3:**
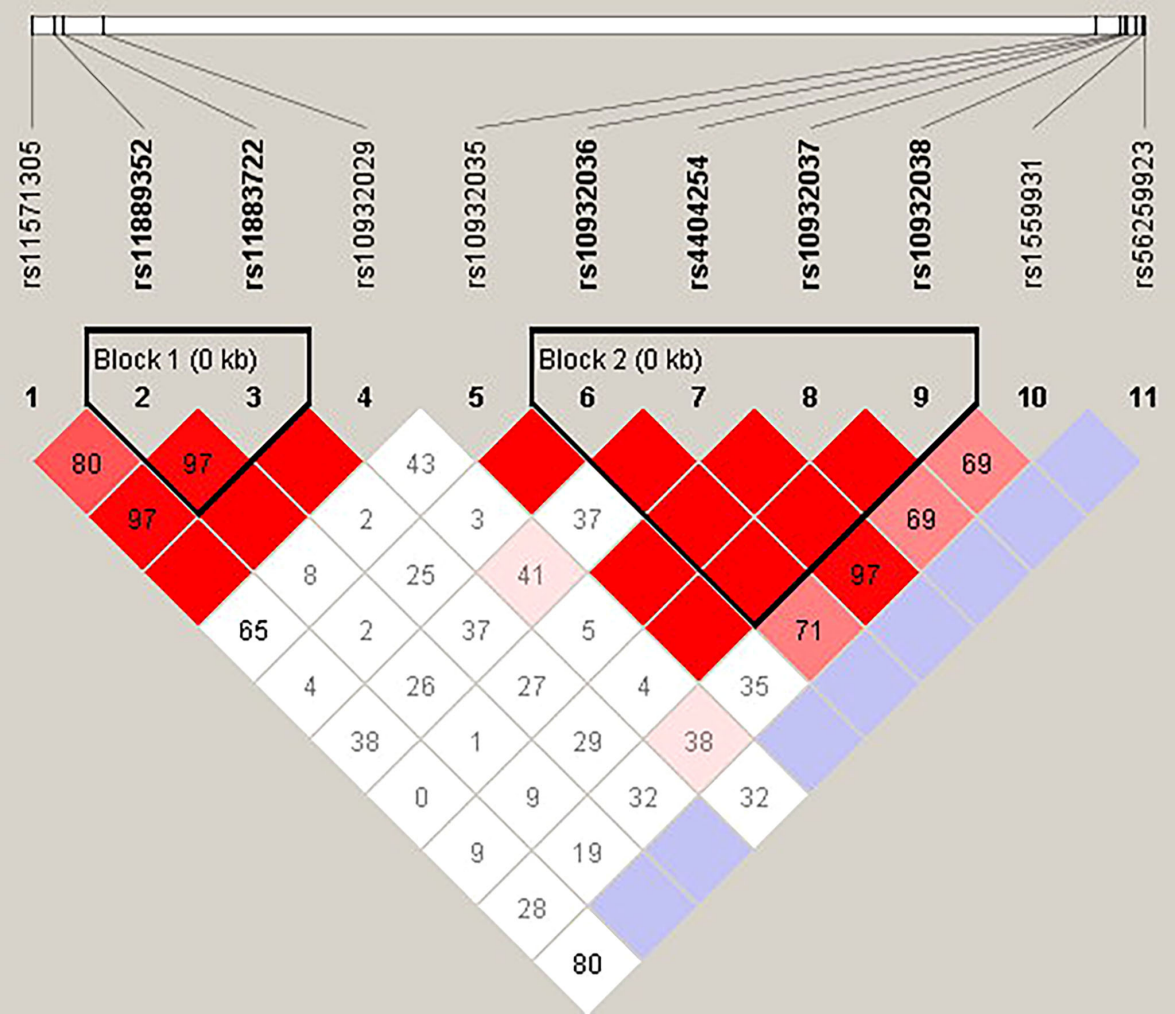
The linkage disequilibrium (LD) analysis of the ICOS gene. There were two haplotype blocks shown in the ICOS gene. One block included rs11889352 and rs11883722, and the other contained rs10932036, rs4404254, rs10932037, and rs10932038.

**Figure 4:**
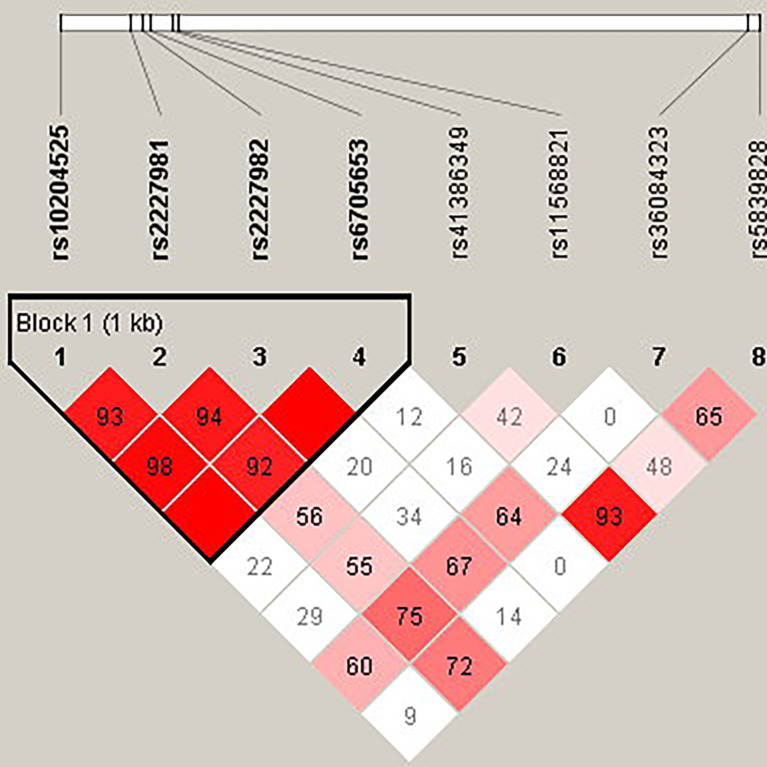
The linkage disequilibrium (LD) analysis of the PDCD1 gene. There was one haplotype block shown in the PDCD1 gene, including rs10204525, rs2227981, rs2227982, and rs6705653.

**Figure 5:**
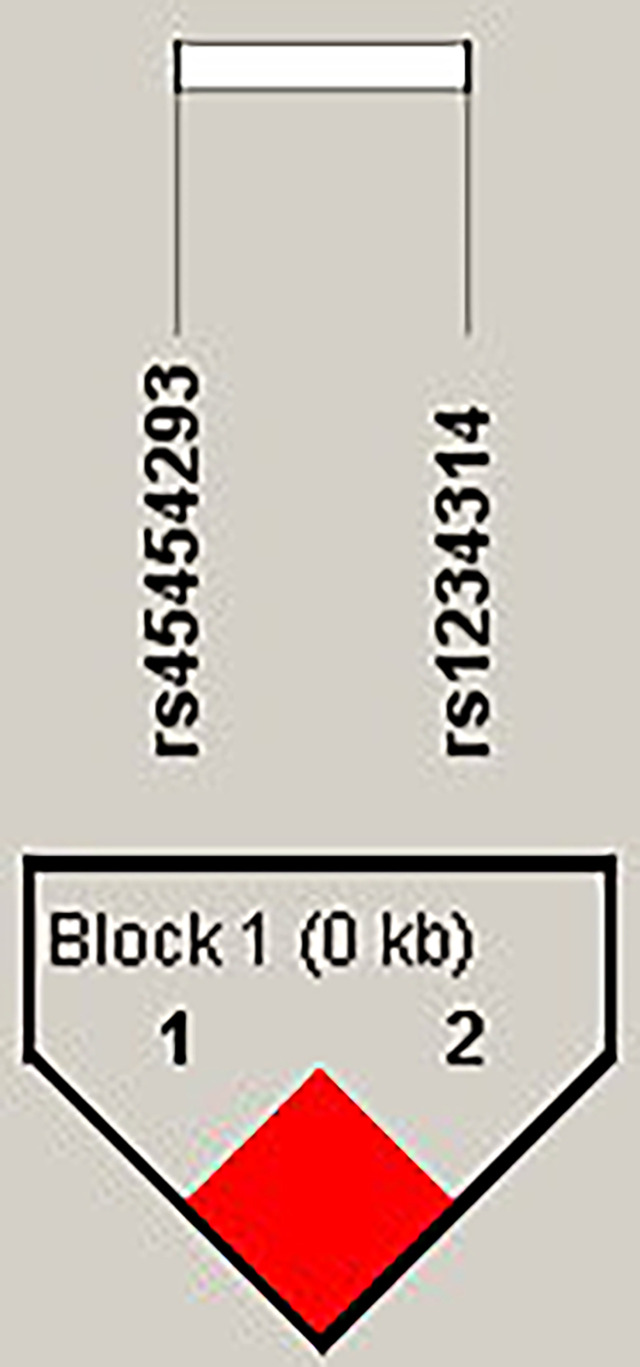
The linkage disequilibrium (LD) analysis of the TNFSF4 gene. There was one haplotype block shown in the TNFSF4 gene, including rs45454293 and rs1234314.

The candidate SNPs of the CTLA4, CD28, PDCD1, ICOS, and TNFSF4 genes were analyzed by LD (**Figures 1**–**5**). The D′ value is shown in the boxes. In the boxes, the red color means the two SNPs have high linkage, and the more the linkage decreases, the closer the color is to white; the light purple color means that they absolutely have no linkage. Haplotype analysis (**Table 4**) showed that CTLA4 Ars62182595Trs16840252 (OR = 0.042, 95% CI = 0.005–0.324, p < 0.001), Ars62182595Crs16840252 (OR = 0.042, 95% CI = 0.005–0.324, p < 0.001), and Grs62182595Trs16840252 (OR = 0.037, 95% CI = 0.005–0.286, p < 0.001), and TNFSF4 Grs1234314Grs45454293 (OR = 0.229, 95% CI = 0.091–0.579, p = 0.001) had a lower risk of SLE. However, the haplotypes in PDCD1, CD28, and ICOS had no significance (**Table 4**).

**Table 4:** Genotype frequencies of the significant SNPs in SLE cases and healthy controls.

Haplotypes	Freq. Cases	Freq. Controls	OR	95% CI.	*p* value
A_rs62182595_T_rs16840252_	0.014	0.253	0.042	0.005-0.324	<0.001
A_rs62182595_C_rs16840252_	0.014	0.253	0.042	0.005-0.324	<0.001
G_rs62182595_T_rs16840252_	0.014	0.280	0.037	0.005-0.286	<0.001
C_rs1234314_C_rs45454293_	0.690	0.907	0.229	0.091-0.579	0.001

Freq., frequency; OR, odds ratio; CI, confidence interval.

## Discussion

According to the results of SNP analysis, we found that there were seven SNPs of the CTLA4 gene, one SNP of the PDCD1 gene, and one SNP of the TNFSF4 gene associated with SLE. Additionally, these SLE-related SNPs also had an association with other autoimmune disorders and cancers. The information is summarized in Supplementary **Table 1**. It is increasingly being appreciated that multiple autoimmune diseases share common susceptibility genes. It was indicated that these SNPs may be a key factor driving immune abnormalities in the immune regulation and pathogenic mechanism of disorders.

For CTLA4, rs11571315 was associated with transfusion reaction (21), polycystic ovary syndrome (22), etc. Yao et al. showed that rs11571315 was significant with the expression level of CTLA4 (23). rs733618 was associated with Graves’ disease (24, 25), non-small cell lung cancer (26), etc. A meta-analysis showed that the allele and genotype frequencies of rs733618 were associated with SLE. Moreover, compared to the CC genotype, people with CT+TT had a lower risk of SLE in an Asian population (27). This was similar to our results. rs4553808 was associated with several autoimmune diseases (28–30), cancers (31, 32), and prognosis post transplantation (33, 34). Wang et al. showed that the G-allele frequency of rs4553808 was higher in breast cancer patients than in controls (35). On the contrary, our result showed that the G-allele frequency was higher in SLE patients than in controls. This result suggested that rs4553808 had opposite effects on different diseases. rs62182595 was also associated with polycystic ovary syndrome (22). rs16840252 was associated with hepatocellular carcinoma (36), antineutrophil cytoplasmic antibody–associated vasculitis (37), rheumatoid arthritis (38), etc., in which rs16840252 had the most significance in haplotype. rs5742909 was associated with cervical cancer (39), schizophrenia (40), chronic liver diseases (41), Hashimoto’s thyroiditis (42), etc. Shojaa et al. showed that rs5742909 was associated with SLE pathogenesis in an Iranian population, where the CC genotype was associated with SLE, while the CT genotype and T allele were more frequent in controls than in SLE cases (43). Our result was similar to theirs. The rs11571319 located in the 3′UTR was associated with rheumatoid arthritis (44), primary biliary cirrhosis (45), asthma (46), etc. the SNPs in the 3′UTR may interfere with mRNA stability and then alter the translation level of proteins (47). 

For PDCD1 and TNFSF4, rs36084323 was associated with cancer risk (48). Ishizaki et al. showed that the rs36084323 G allele had a significantly higher promoter activity than the A allele (49). rs1234314 was associated with allergic rhinitis (50), coronary artery disease (51), etc. It was shown that the CC genotype of rs1234314 provided a protective effect against allergic rhinitis (50), which was the same as our result. These SNPs were all located in the non-coding region. Thus, they may affect the immunity by altering the expression of the gene. Additionally, the results showed that most of the significant SNPs were in the CTLA4 gene. This may be because CTLA4 is mainly expressed on Tregs. Moreover, the autoreactive T-cell response was strictly controlled by Tregs in the periphery. The abnormality of any one of the co-stimulatory/co-inhibitory signals would lead to these autoreactive T cells’ uncontrolled expansion, causing the development of AD (52). Moreover, haplotype analysis showed that A_rs62182595_T_rs16840252_, A_rs62182595_C_rs16840252_, G_rs62182595_T_rs16840252_, and G_rs1234314_G_rs45454293_ haplotypes may decrease the risk of SLE. This suggested the possibility of an interaction between the two SNPs in one haplotype in SLE susceptibility, in view of the high LD between polymorphisms.

Abnormal expression and function of co-stimulatory/co-inhibitory molecules have been described to be associated with aberrant T-cell activation in SLE patients, which results in reduction in the T-cell activation threshold and loss of peripheral immune tolerance (53). These data further advance our understanding of the complex immunopathogenesis of SLE and provide additional support for the emerging concept of shared genes in multiple autoimmune diseases.

In summary, our results showed that several SNPs of immune regulatory genes were significant with SLE, which were almost located in non-coding regions. It should be further verified whether SNP variations affect gene expression or protein function. Moreover, epigenetic markers can also affect the expression level of a gene, leading to abnormal CD4+ T-cell function, which in turn increases the risk of autoimmune disease. Consequently, epigenetic markers should be considered together in the future.

### Limitation

The co-stimulatory system is involved in the regulation of T-cell activation. The expression level of co-stimulatory/co-inhibitory molecules will affect the degree of T-cell activation. Therefore, we focused on the promoter region of genes. Due to limited funds, we searched the literature related to autoimmune diseases and selected the hot SNPs related to autoimmune diseases as candidate SNPs for discussion. We explored the 500-bp flanking region of the candidate SNPs, especially on the promoter region, rather than whole-genome region. In addition, because of insufficient genomic DNA and failure of PCR reaction, not every sample had complete SNP data available.

## Data availability statement

The original contributions presented in the study are included in the article/**Supplementary Material**. Further inquiries can be directed to the corresponding author.

## Ethics statement

The Institutional Review Board of Chang Gung Memorial Hospital has reviewed and approved the study. The approval ID was 202002097B0. The patients/participants provided their written informed consent to participate in this study.

## Author contributions

D-PC conceived and designed the experiments and reviewed the final draft. W-TL performed the experiments and analyzed and interpreted the data. K-HY wrote the draft of the manuscript and provided the samples. All authors read and approved the final manuscript.

## Funding

This study was supported by grants to D-PC from the Ministry of Science and Technology (110-2320-B-182A-007). The funders had no role in the study design, data collection and analysis, decision to publish, or preparation of the manuscript.

## Conflict of interest

The authors declare that the research was conducted in the absence of any commercial or financial relationships that could be construed as a potential conflict of interest.

## Publisher’s note

All claims expressed in this article are solely those of the authors and do not necessarily represent those of their affiliated organizations, or those of the publisher, the editors and the reviewers. Any product that may be evaluated in this article, or claim that may be made by its manufacturer, is not guaranteed or endorsed by the publisher.
