# Corrigendum: Investigation of the association between the genetic polymorphisms of co-stimulatory system and systemic lupus erythematosus

**DOI:** 10.3389/fimmu.2022.1113152

**Published:** 2023-01-16

**Authors:** Ding-Ping Chen, Wei-Tzu Lin, Kuang-Hui Yu

**Affiliations:** ^1^ Department of Laboratory Medicine, Linkou Chang Gung Memorial Hospital, Taoyuan, Taiwan; ^2^ Department of Medical Biotechnology and Laboratory Science, College of Medicine, Chang Gung University, Taoyuan, Taiwan; ^3^ Division of Rheumatology, Allergy, and Immunology, Chang Gung University and Memorial Hospital, Taoyuan, Taiwan

**Keywords:** systemic lupus erythematosus (SLE), co-stimulatory/co-inhibitory molecules, single nucleotide polymorphism (SNP), autoimmune disease (AD), association

In the published article, there was an error. The TNFSF4 gene is reverse, in which the wild-type allele of rs1234314 is C rather than G and the wild-type allele of rs45454293 is C rather than G. Originally, the direction of the primer was wrong, so the allele on the sequence we read was followed by the error. Because the complementary base of C is G and rs45454293 is exactly C to G mutation, we didn’t find this error at that time. This mistake will cause the opposite result, leading to a misunderstanding about risk allele for SLE. Thus, there are 5 errors in the original manuscript needed to be corrected.

1. A correction has been made to the **Abstract**.

This sentence previously stated: “GG vs. CC: p=0.004; GG+ CG vs. CC: p=0.001”

The corrected sentence appears below: “CC vs. GG: p=0.004; CC+ CG vs. GG: p=0.001”.

2. A correction has been made to **Results**, *The analysis of genotype frequencies*.

This sentence previously stated: “Compared to the GG genotype, the subjects with the CC genotype would have a 4.4 times risk of SLE (95% CI =1.577-12.275, p = 0.004), which also had significance based on the dominant model (GG+CG vs. CC: OR = 4.362, 95% CI = 1.727-11.015, p = 0.001)”.

The corrected sentence appears below: “Compared to the CC genotype, the subjects with the GG genotype would have a 4.4 times risk of SLE (95% CI =1.577-12.275, p = 0.004), which also had significance based on the dominant model (CC+CG vs. GG: OR = 4.362, 95% CI = 1.727-11.015, p = 0.001)”.

3. A correction has been made to **Discussion**, paragraph 3. This sentence previously stated:

“It was shown that the CC genotype of rs1234314 provided a protective effect against allergic rhinitis (50), which was contrary to our result”.

The corrected sentence appears below:

“It was shown that the CC genotype of rs1234314 provided a protective effect against allergic rhinitis (50), which was the same as our result”.

4. In the published article, there was an error in **Table 2** as published. The “SNP” and “Allele” columns of the TNFSF4 gene section previously contained the values “rs1234314 **C**/G and rs45454293 **A**/G” when they should contain the values “rs1234314 G/**C** and rs45454293 C/**T**”. The corrected **Table 2** and its caption appears below.

**Table 2:** The HWE analysis in control group and the allele frequencies in cases and controls.

SNP	Position	Allele	Minor allele frequency		HWE *p* value	Odds ratio	*p^a^* value
			Patient	Control		(95%CI)	
CTLA4							
rs11571315	203866178	**C**/T	0.148	0.353	0.710	0.318 (0.179-0.563)	<0.001*
rs733618	203866221	T/**C**	0.417	0.573	0.817	0.532 (0.335-0.845)	0.007*
rs4553808	203866282	A/**G**	0.007	0.133	0.412	0.045 (0.006-0.343)	<0.001*
rs11571316	203866366	**A**/G	0.157	0.220	0.654	0.661 (0.364-1.201)	0.172
rs62182595	203866465	**A**/G	0.007	0.133	0.946	0.047 (0.006-0.353)	<0.001*
rs16840252	203866796	C/**T**	0.021	0.147	0.330	0.126 (0.037-0.430)	<0.001*
rs5742909	203867624	C/**T**	0.079	0.140	0.370	0.524 (0.243-1.130)	0.095
rs231775	203867991	**A**/G	0.325	0.349	0.999	0.899 (0.542-1.488)	0.678
rs3087243	203874196	G/**A**	0.239	0.227	0.752	1.013 (0.589-1.740)	0.964
rs11571319	203874215	G/**A**	0.132	0.280	0.814	0.358 (0.196-0.655)	0.001*
CD28							
rs1879877	203705277	G/**T**	0.466	0.456	0.991	1.083 (0.684-1.714)	0.733
rs3181096	203705369	C/**T**	0.247	0.284	0.220	0.826 (0.494-1.383)	0.468
rs3181097	203705416	G/**A**	0.419	0.419	0.895	1.000 (0.630-1.587)	1.000
rs3181098	203705655	G/**A**	0.277	0.258	0.164	1.109 (0.662-1.857)	0.693
rs56228674	203729436	C/**T**	0.033	0.040	0.979	0.828 (0.155-4.405)	1.000
rs3116496	203729789	T/**C**	0.107	0.120	0.793	0.876 (0.323-2.376)	0.794
PDCD1							
rs5839828	241859601	**G**/GG	0.338	0.289	0.868	1.258 (0.761-2.079)	0.371
rs36084323	241859444	**C**/T	0.493	0.317	0.997	2.096 (1.293-3.397)	0.003*
rs41386349	241851697	G/**A**	0.222	0.180	0.470	1.302 (0.734-2.308)	0.366
rs6705653	241851407	**T**/C	0.285	0.216	0.572	1.443 (0.847-2.459)	0.177
rs2227982	241851281	**G**/A	0.471	0.392	0.953	1.384 (0.867-2.210)	0.173
rs2227981	241851121	**A**/G	0.261	0.223	0.297	1.232 (0.713-2.127)	0.454
rs10204525	241850169	**C**/T	0.250	0.207	0.990	1.280 (0.642-2.552)	0.483
ICOS							
rs11571305	203935403	G/**A**	0.297	0.336	0.007*	0.836 (0.504-1.388)	0.489
rs11889352	203935948	**T**/A	0.254	0.243	0.126	1.059 (0.617-1.818)	0.836
rs11883722	203936122	G/**A**	0.418	0.421	0.491	0.985 (0.616-1.576)	0.951
rs10932029	203937045	T/**C**	0.164	0.110	0.350	1.586 (0.789-3.188)	0.193
rs10932035	203959929	**G**/A	0.463	0.500	<0.001*	0.833 (0.486-1.430)	0.508
rs10932036	203960458	**A**/T	0.047	0.056	0.154	0.844 (0.288-2.475)	0.757
rs4404254	203960563	T/**C**	0.192	0.269	0.995	0.646 (0.368-1.133)	0.126
rs10932037	:203960623	C/**T**	0.034	0.082	0.673	0.397 (0.134-1.173)	0.085
rs10932038	203960861	A/**G**	0.035	0.077	0.561	0.432 (0.144-1.298)	0.125
rs1559931	203961006	G/**A**	0.197	0.227	0.980	0.838 (0.467-1.505)	0.555
rs56259923	203961015	G/**T**	0.014	0.016	0.992	0.900 (0.125-6.484)	1.000
rs4675379	203961372	G/**C**	0.156	0.161	0.598	0.967 (0.393-2.381)	0.942
TNFSF4							
rs1234314	173208253	G/**C**	0.514	0.360	0.395	1.881 (1.177-3.005)	0.008*
rs45454293	173208097	C/**T**	0.148	0.160	0.998	0.911 (0.482-1.722)	0.774

The position was obtained from Genome Assembly GRCh38.p13. rs: reference SNP; HWE: Hardy-Weinberg equilibrium; 95% CI: 95% confidence interval; P^a^ values of allele frequency were counted from Chi-square test or Fisher’s exact test. In the column of “Allele”, the bold was referred to minor allele, and the minor allele was referred to the allele with lower frequency in the population containing cases and controls. “*” was expressed as p<0.05.

5. In the published article, there was an error in **Table 3** as published. The “Genotype” column of the TNFSF4 gene section had “GG” and “CC” in the wrong positions. The corrected **Table 3** and its caption appears below.

**Table 3:** Genotype frequencies of the significant SNPs in SLE cases and healthy controls.

SNP	Genotype	Genotype frequency		Odds ratio 95% CI.	*p* value
		Patient	Control		
CTLA4					
rs11571315	CC vs. CT vs. TT				0.001*
	TT	53	33	Ref.	1.000
	CT	15	31	0.301 (0.142-0.641)	0.001
	CC	3	11	0.170 (0.044-0.654)	0.005**
	TT vs. CT + CC			0.267 (0.132-0.539)	<0.001*
	TT + CT vs. CC			0.257 (0.068-0.962)	0.032*
rs733618	CC vs. CT vs. TT				0.002*
	CC	33	15	Ref.	1.000
	CT	18	34	0.241 (0.104-0.555)	0.001*
	TT	21	26	0.367 (0.159-0.849)	0.018*
	CC vs. CT + TT			0.295 (0.142-0.614)	0.001*
	CC + CT vs. TT			0.776 (0.387-1.556)	0.475
rs4553808	AA vs. AG vs. GG				<0.001*
	AA	71	55	Ref.	1.000
	AG	1	20	0.039 (0.005-0.298)	<0.001*
	GG	0	0	NA	NA
	AA vs. AG+GG			0.039 (0.005-0.298)	<0.001*
	AA+AG vs. GG			NA	NA
rs62182595	GG vs. AG vs. AA				<0.001*
	GG	69	56	Ref.	1.000
	AG	1	18	0.045 (0.006-0.348)	<0.001**
	AA	0	1	NA	0.452
	GG vs. AG+AA			0.043 (0.006-0.329)	<0.001*
	GG+AG vs. AA			NA	1.000
rs16840252	CC vs. CT vs. TT				<0.001*
	CC	69	53	Ref.	1.000
	CT	1	22	0.035 (0.005-0.267)	<0.001*
	TT	1	0	NA	1.000
	CC vs. CT + TT			0.070 (0.016-0.310)	<0.001*
	CC + CT vs. TT			NA	0.486
rs5742909	CC vs. CT vs. TT				0.051
	CC	60	54	Ref.	1.000
	CT	9	21	0.386 (0.163-0.914)	0.027*
	TT	1	0	NA	1.000
	CC vs. CT + TT			0.429 (0.185-0.991)	0.044*
	CC + CT vs. TT			NA	0.493
rs11571319	GG vs. AG vs. AA				<0.001*
	GG	58	40	Ref.	1.000
	AG	2	28	0.049 (0.011-0.219)	<0.001*
	AA	8	7	0.788 (0.265-2.348)	0.669
	GG vs. AG+AA			0.197 (0.088-0.443)	<0.001*
	GG+AG vs. AA			1.295 (0.443-3.784)	0.636
PDCD1					
rs36084323	CC vs. CT vs. TT				0.013*
	TT	19	33	Ref.	1.000
	CT	34	31	1.905 (0.904-4.014)	0.089
	CC	18	7	4.466 (1.579-12.631)	0.004*
	TT vs. CT+CC			2.377 (1.177-4.798)	0.015*
	TT+CT vs. TT			3.105 (1.206-7.996)	0.015*
TNFSF4					
rs1234314	CC vs. CG vs. GG				0.005*
	CC	20	28	Ref.	1.000
	CG	29	40	1.015 (0.481-2.142)	0.969
	GG	22	7	4.400 (1.577-12.275)	0.004*
	CC vs. CG+ GG			1.519 (0.756-3.051)	0.239
	CC+ CG vs.GG			4.362 (1.727-11.015)	0.001*

95% CI, 95% confidence interval; NA, not applicable. “*” was expressed as p<0.05.

6. In the published article, there was an error in **Table 4** as published. The last row of the “Haplotypes” column previously contained “G”s instead of “C”s.

**Table 4:** Genotype frequencies of the significant SNPs in SLE cases and healthy controls.

Haplotypes	Freq. Cases	Freq. Controls	OR	95% CI.	*p* value
A_rs62182595_T_rs16840252_	0.014	0.253	0.042	0.005-0.324	<0.001
A_rs62182595_C_rs16840252_	0.014	0.253	0.042	0.005-0.324	<0.001
G_rs62182595_T_rs16840252_	0.014	0.280	0.037	0.005-0.286	<0.001
C_rs1234314_C_rs45454293_	0.690	0.907	0.229	0.091-0.579	0.001

Freq., frequency; OR, odds ratio; CI, confidence interval.

The corrected **Table 4** and its caption appears below.

The authors apologize for this error and state that this does not change the scientific conclusions of the article in any way. The original article has been updated.

