# Supplementary material for: Investigation of the association between the genetic polymorphisms of the co-stimulatory system and systemic lupus erythematosus
Source: Front Immunol. 2022 Sep 6;13:946456. doi: 10.3389/fimmu.2022.946456 (PMC9521740; doi:10.3389/fimmu.2022.946456)
Supplement: Supplementary file 1 [file DataSheet_1.docx]

Supplementary Material

**Supplementary Figure 1.** Summarized the diseases that were associated with the significant SNPs in this study.

| **SNP** | **disease** | **ref** |
| --- | --- | --- |
| rs11571315 | transfusion reaction  polycystic ovary syndrome | [1]  [2] |
| rs733618 | Graves’ ophthalmopathy  Graves’ disease  non-small cell lung cancer | [3]  [4]  [5] |
| rs4553808 | Hashimoto's thyroiditis disease  multiple sclerosis  type 1 diabetes  breast cancer and head and neck cancer in Asian population  gastric cancer  viral infection in kidney transplantation  relapse-free survival and OS after allo-HSCT. | [6]  [7]  [8]  [9]  [10]  [11]  [12] |
| rs62182595 | polycystic ovary syndrome | [2] |
| rs16840252 | Graves’ ophthalmopathy (haplotype)  hepatocellular carcinoma (haplotype)  antineutrophil cytoplasmic antibody (ANCA)–associated vasculitis  rheumatoid arthritis | [3]  [13]  [14]  [15] |
| rs5742909 | cervical cancer  schizophrenia  chronic liver diseases  Hashimoto's thyroiditis | [16]  [17]  [18]  [19] |
| rs11571319 | rheumatoid arthritis  primary biliary cirrhosis  asthma | [20]  [21]  [22] |
| rs36084323 | cancer risk | [23] |
| rs1234314 | allergic rhinitis  coronary artery disease | [24]  [25] |

Reference

1. Wen, Y.H., Lin, W.T., Wang, W.T., Chiueh, T.S., and Chen, D.P. (2019) Association of CTLA4 gene polymorphism with transfusion reaction after infusion of leukoreduced blood component. J Clin Med. 8:1961. doi: 10.3390/jcm8111961.
2. Abdul-jabbar, R.A.-a. (2021). Significance of CTL4 gene polymorphisms in susceptibility to polycystic ovary syndrome of Iraqi women. Annals of R.S.C.B. 25:6624-32.
3. Chen, D.P., Chu, Y.C., Wen, Y.H., Lin, W.T., Hour, A.L., and Wang, W.T. (2019). Investigation of the correlation between graves' ophthalmopathy and CTLA4 gene polymorphism. J Clin Med. 8:1842. doi: 10.3390/jcm8111842.
4. Chen, P.L., Fann, C.S., Chang, C.C., Wu, I.L., Chiu, W.Y., Lin, C.Y., et al. (2008). Family-based association study of cytotoxic T-lymphocyte antigen-4 with susceptibility to Graves' disease in Han population of Taiwan. Genes Immun. 9:87-92. doi: 10.1038/sj.gene.6364445.
5. Chen, S., Wang, Y., Chen, Y., Lin, J., Liu, C., Kang, M., et al. (2017). Investigation of Cytotoxic T-lymphocyte antigen-4 polymorphisms in non-small cell lung cancer: a case-control study. Oncotarget. 8:76634-76643. doi: 10.18632/oncotarget.20638.
6. Kaykhaeia, M., Moghadamb, H., Dabiric, S., Salimidg, S., Jahantighb, D., Tamandani, D.M.K., et al. (2020). Association of CTLA4 (rs4553808) and PTPN22 (rs2476601) gene polymorphisms with Hashimoto's thyroiditis disease: A case-control study and an In-silico analysis. Meta Gene. 24:100693. doi: 10.1016/j.mgene.2020.100693
7. Yousefipour, G., Erfani, N., Momtahan, M., Moghaddasi, H., and Ghaderi, A. (2009). CTLA4 exon 1 and promoter polymorphisms in patients with multiple sclerosis. Acta Neurol Scand. 120:424-9. doi: 10.1111/j.1600-0404.2009.01177.x.
8. Bouqbis, L., Izaabel, H., Akhayat, O., Pérez-Lezaun, A., Calafell, F., Bertranpetit, J., et al. (2003). Association of the CTLA4 promoter region (-1661G allele) with type 1 diabetes in the South Moroccan population. Genes Immun. 4:132-7. doi: 10.1038/sj.gene.6363933
9. Fang, M., Huang, W., Mo, D., Zhao, W., and Huang, R. (2018). Association of five SNPs in cytotoxic T-lymphocyte antigen 4 and cancer susceptibility: evidence from 67 studies. Cell Physiol Biochem. 47:414-427. doi: 10.1159/000489953.
10. Li, J., Wang, W., Sun, Y., and Zhu, Y. (2020). CTLA-4 polymorphisms and predisposition to digestive system malignancies: a meta-analysis of 31 published studies. World J Surg Oncol. 18:55. doi: 10.1186/s12957-020-1806-2.
11. Guo, Y., Guo, F., Wei, C., Qiu, J., Liu, Y., Fang, Y., et al. (2013). CTLA4 gene polymorphisms influence the incidence of infection after renal transplantation in Chinese recipients. PLoS One. 8:e70824. doi: 10.1371/journal.pone.0070824.
12. Jagasia, M., Clark, W.B., Brown-Gentry, K.D., Crawford, D.C., Fan, K.H., Chen, H., et al. (2012). Genetic variation in donor CTLA-4 regulatory region is a strong predictor of outcome after allogeneic hematopoietic cell transplantation for hematologic malignancies. Biol Blood Marrow Transplant. 18:1069-75. doi: 10.1016/j.bbmt.2011.12.518.
13. Yang, J., Liu, J., Chen, Y., Tang, W., Liu, C., Sun, Y., et al. (2019). Association of CTLA-4 tagging polymorphisms and haplotypes with hepatocellular carcinoma risk: A case-control study. Medicine (Baltimore). 98:e16266. doi: 10.1097/MD.0000000000016266.
14. Lyons, P.A., Rayner, T.F., Trivedi, S., Holle, J.U., Watts, R.A., Jayne, D.R., Baslund, B., et al. (2012). Genetically distinct subsets within ANCA-associated vasculitis. N Engl J Med. 367:214-23. doi: 10.1056/NEJMoa1108735..
15. Liu, W., Yang, Z., Chen, Y., Yang, H., Wan, X., Zhou, X., et al. The association between CTLA-4, CD80/86, and CD28 gene polymorphisms and rheumatoid arthritis: an original study and meta-analysis. Front Med (Lausanne). 8:598076. doi: 10.3389/fmed.2021.598076.
16. Hu, S., Pu, D., Xia, X., Guo, B., and Zhang, C. (2020). CTLA-4 rs5742909 polymorphism and cervical cancer risk: A meta-analysis. Medicine (Baltimore). 99:e19433. doi: 10.1097/MD.0000000000019433..
17. Sumirtanurdin, R., Laksono, J.P., Dania, H., Ramadhani, F.N., Perwitasari, D.A., Abdulah, R., et al. (2019). Single-nucleotide Polymorphism of CTLA-4 (rs5742909) in Correlation with Schizophrenia Risk Factor. J Pharm Bioallied Sci. 11:S605-S610. doi: 10.4103/jpbs.JPBS_215_19.
18. Zhang, S., Yang, X., and Wang, W. (2020). Associations of genetic polymorphisms in CTLA-4 and IL-18 with chronic liver diseases: Evidence from a meta-analysis. Genomics. 112:1889-96. doi: 10.1016/j.ygeno.2019.11.001.
19. Narooie-Nejad, M., Taji, O., Kordi Tamandani, D.M, and Kaykhaei, M.A. (2017). Association of CTLA-4 gene polymorphisms -318C/T and +49A/G and Hashimoto's thyroidits in Zahedan, Iran. Biomed Rep. 6:108-12. doi: 10.3892/br.2016.813.
20. Aslam, M.M., Jalil, F., John, P., Fan, K.H., Bhatti, A., Feingold, E., et al. (2020). A sequencing study of CTLA4 in Pakistani rheumatoid arthritis cases. PLoS One. 15:e0239426. doi: 10.1371/journal.pone.0239426.
21. Juran, B.D., Atkinson, E.J., Schlicht, E.M., Fridley, B.L., and Lazaridis, K.N. (2008). Primary biliary cirrhosis is associated with a genetic variant in the 3' flanking region of the CTLA4 gene. Gastroenterology. 135:1200-6. doi: 10.1053/j.gastro.2008.06.077.
22. Choi, H., Tabashidze, N., Rossner, P. Jr., Dostal, M., Pastorkova, A., Kong, S.W., et al. (2017). Altered vulnerability to asthma at various levels of ambient Benzo[a]Pyrene by CTLA4, STAT4 and CYP2E1 polymorphisms. Environ Pollut. 231:1134-1144. doi: 10.1016/j.envpol.2017.07.057.
23. Da, L.S., Zhang, Y., Zhang, C.J., Bu, L.J., Zhu, Y.Z., Ma, T., et al. (2018). The PD-1 rs36084323 A > G polymorphism decrease cancer risk in Asian: A meta-analysis. Pathol Res Pract. 214:1758-1764. doi: 10.1016/j.prp.2018.09.015.
24. Shen, Y., Liu, Y., Wang, X.Q., Ke, X., Kang, H.Y., Hong, S.L. (2017). Association between TNFSF4 and BLK gene polymorphisms and susceptibility to allergic rhinitis. Mol Med Rep. 16:3224-3232. doi: 10.3892/mmr.2017.6954.
25. Liu, S., Wang, X., Yu, S., Yan, M., Peng, Y., Zhang, G., et al. (2020). A meta-analysis on the association between TNFSF4 polymorphisms (rs3861950 T > C and rs1234313 A > G) and susceptibility to coronary artery disease. Front Physiol. 11:539288. doi: 10.3389/fphys.2020.539288.
